# Expression of the receptor of advanced glycation end-products (RAGE) and membranal location in peripheral blood mononuclear cells (PBMC) in obesity and insulin resistance

**DOI:** 10.22038/ijbms.2019.34571.8206

**Published:** 2019-06

**Authors:** Elizabeth del Carmen Ruelas Cinco, Bertha Ruíz Madrigal, José Alfredo Domínguez Rosales, Montserrat Maldonado González, Lucía De la Cruz Color, Sandra Margarita Ramírez Meza, José Rodrigo Torres Baranda, Erika Martínez López, Zamira Helena Hernández Nazará

**Affiliations:** 1 Instituto de Investigación en Enfermedades Crónico-Degenerativas, Departamento de Biología Molecular y Genómica, C.U.C.S, Universidad de Guadalajara, Guadalajara, Jalisco, México; 2 Programa de Doctorado en Ciencias en Biología Molecular en Medicina. Departamento de Biología Molecular y Genómica, C.U.C.S, Universidad de Guadalajara, Guadalajara, Jalisco, México; 3 Laboratorio de Investigación en Microbiología, Departamento de Microbiología y Patología, C.U.C.S, Universidad de Guadalajara, Guadalajara, Jalisco, México

**Keywords:** AGER protein human, Insulin resistance, Obesity, Oxidative stress, Receptor for advanced - glycation end products

## Abstract

**Objective(s):**

The present study aimed to evaluate the receptor of advanced glycation end-products (RAGE), NF-kB, NRF2 gene expression, and RAGE cell distribution in peripheral blood mononuclear cells (PBMC) in subjects with obesity and IR compared with healthy subjects.

**Materials and Methods:**

The mRNA expression levels of RAGE, NF-kB, NRF2, and GAPDH were determined in PBMC by qPCR in 20 obese (OB), 17 obese with insulin resistance (OB-IR), and 20 healthy subjects (HS), matched by age and sex. RAGE protein expression and its localization were determined by Western Blot and immunocytochemistry (ICC) analysis, total soluble RAGE (sRAGE) and MCP-1 plasma levels by ELISA.

**Results::**

RAGE, NF-kB, and NRF2 genes mRNA expression in PBMCs did not show variation between groups. RAGE protein was lower in OB and OB-IR groups; RAGE was located predominantly on the cell-surface in the OB-IR group compared to the HS group (22% vs 9.5%, *P*<0.001). OB-IR group showed lower sRAGE plasma levels, and correlated negatively with HOMA-IR, ALT parameters (r= -0.374, r= -0.429, respectively), and positively with *NFE2L2* mRNA (r= 0.540) *P*<0.05.

**Conclusion:**

In this study, OB-IR subjects did not reflect significant differences in gene expression; however, correlations detected between sRAGE, biochemical parameters, and NRF2, besides the predominant RAGE distribution on the cell membrane in PBMC could be evidence of the early phase of the inflammatory cascade and the subsequent damage in specific tissues in subjects with OB-IR.

## Introduction

Obesity has been increasing around the world, and it is considered a risk factor for the development of metabolic alterations such as insulin resistance (IR), type 2 diabetes (T2D), cardiovascular disease, and cancer (1). IR is hard to detect as an early phase of chronic disease by clinical screening because of the maintenance of blood glucose <125 mg/dl, despite impairing glycemia. However, when beta cells in the pancreas are not able to maintain insulin hypersecretion, and it begins to deteriorate, the failure of insulin release is the point of diagnosis in most cases of Metabolic Syndrome (MetS) and T2D. The aforementioned represents a major social problem with devastating long-term consequences in Mexico, which is one of the countries with the highest prevalence and incidence of obesity worldwide (1, 2). In this context, we believe that the timely detection of obesity before complications arise is critical, especially when this can be prevented with changes in lifestyle.

The increase in reactive oxygen species (ROS) and exacerbation of cytokines appear to be a deleterious factor that is linked to disorders like obesity and IR. The receptor for advanced glycation end products (RAGE) was associated with the risk of MetS (3). Peripheral blood mononuclear cells (PBMC) seem to be good sensors of the environmental effects and the metabolic state (4). RAGE expression in PBMC, being a ubiquitous multiligand of the immunoglobulin superfamily of transmembrane receptors, could be the key mediator of the inflammatory/oxidative state in obesity.

Different RAGE isoforms have been identiﬁed by alternative splicings, such as RAGE full-length (RAGE-fl), the complete and major isoform of RAGE**; **N-terminal truncated (Nt-RAGE), which lacks the type V extracellular domain**;** the dominant-negative RAGE (DN-RAGE), which lacks the cytosolic domain, and endogenous secretory RAGE (esRAGE), the latter lacks cytosolic and transmembrane domains, therefore, is free in the blood circulation. Also, RAGE suffers a proteolytic cut that releases it into circulation and is known as soluble RAGE (sRAGE) (5, 6). RAGE-ligand interaction results in a rapid and sustained cellular activation of NFκB, accompanied by increased expression of the receptor itself (7). The increased production of free radicals, consequential of the transcriptional activation of NFκB, is crucial for the activation of NRF2 (8), a key regulator of antioxidant signaling.

Therefore, in the present work, we analyzed the gene expression of *AGER* (RAGE), *RELA* (NF-kB p65sub-unity), and *NFE2L2* (NRF2) in PBMC of obese individuals with and without insulin resistance.

## Materials and Methods

***Subjects***

The study groups consisted of 20 lean healthy-subjects (BMI 18–24.9), 20 obese (BMI 30–39.9), and 17 obese with IR subjects. Individuals were selected through stratified random sampling in order to ensure age and sex-ratio matched.

The Local Ethics Committee of the Centro Universitario de Ciencias de la Salud of the Universidad de Guadalajara, Mexico (CI/027/2015) approved this protocol, and we carried it out according to the Declaration of Helsinki (9). All participants gave their written informed consent before their inclusion in this study.

Inclusion and exclusion criteria were based on anthropometric, biochemistry, and clinical data; exclusion criteria included incomplete data, alcohol drinkers (>20 mg/dl per day), smokers (at least one cigarette per day), pregnant women, acute infection, chronic and autoimmune diseases, individuals using glucose/lipid-lowering drugs, and hormonal and anti-inflammatory therapies.

***Study procedures***

*Anthropometric parameters*

Body weight and composition were determined using tetrapolar body electrical bioimpedance (InBody 3.0, Biospace Co, Ltd, South Korea). BMI was calculated as weight in kilograms divided by the height in meters squared. The waist (the narrowest diameter between the lowest borders of the rib cage and the iliac crest), and hip (the widest portion of buttocks) circumferences were measured to calculate the waist-hip index.

*Biochemical parameters*

Blood samples were collected after a 12-hr fasting period and processed on the same day. Biochemical parameters were performed by dry chemistry on a VITROS® 250 Analyzer (Johnson & Johnson, Rochester, NY, USA). Serum aliquots were stored at -20 ^°^C to perform the enzyme-linked immunoassay.

*Analysis of insulin, sRAGE, MCP-1, glycated hemoglobin, and high sensitivity-C reactive protein*

Insulin levels (Monobind Inc, CA, USA; Cat. DA-2425300) were measured to determine IR by the homeostasis model assessment (HOMA-IR) index as follows: HOMA-IR index = (fasting insulin [μU/mL] × fasting glucose [mg/dl]) divided by 405. A HOMA-IR>2.5 was considered an indicator of IR according to Matthews *et al. *(10) and supported based on the guidelines by NCEP/ATP III.

Serum levels of total sRAGE and CCL2/MCP-1 were determined by ELISA following the manufacturer’s instructions (Quantikine® Human, R&D Systems, USA; Cat. DRG00 / DCP00, respectively). The minimum detectable points were 15.821 pg/mL for total sRAGE and 9.986 pg/mL for MCP-1. The coefficient of variation for the assays was less than 10%. 

The glycated hemoglobin (HbA1c) values in 1 mL of EDTA anticoagulated blood samples and *high sensitivity-C reactive protein (*hs-CRP) concentrations in serum were measured using an immunoturbidimetric assay (BioSystems SA, Spain; Cat. 13044 / 31927, respectively) and determined by spectrometry in an ELISA microplate reader following the manufacturer’s instructions. The measurement limit of hs-CRP was 0.7 mg/l. Analyses were performed at least in duplicate and case and controls were placed proportionally in microplates.

*PBMC isolation*

Six to eight milliliters of venous blood samples were collected from each patient into tubes containing EDTA. PBMC were isolated by density gradient centrifugation using Lymphoprep TM (Axis-Shield, Norway; Cat. AXS-1114547 6). The cell pellet was ready for RNA extraction**,** and cell smear was performed for immunocytochemistry (ICC). Some aliquots were stored at -80 ^°^C to perform protein extraction. All these procedures were done within the two hours following blood collection.

*RNA extraction and cDNA synthesis*

Total RNA was isolated from PBMC by the single-step method reported by Chomczynski and Sacchi (TRIzol, ThermoFisher Scientific Inc., USA; Cat. 15596026). The quantity and purity of isolated RNA samples were estimated using a P-Class nanophotometer (IMPLEN Inc. Germany). The Integrity of the ribosomal RNA bands was assessed by 1.5% agarose gel electrophoresis. Reverse transcription was performed on 1 µg of high-quality RNA to produce cDNA in a reaction volume of 20 µl using 10 U/µl of *Moloney murine leukemia virus* reverse transcriptase containing 1× PCR buffer and 0.5 U/µl RNA inhibitor (Invitrogen Life Technologies, USA; Cat. 28025-013 / 10777019, respectively) and then stored at -20 ^°^C until analysis.

*Quantitative PCR*

qPCR was performed to analyze *AGER*,* RELA*,* NFE2L2*, and *GAPDH* mRNA gene expression. Oligonucleotide primer pairs and their suitable probes were designed using the UPL Assay Design Center web service (*http://qpcr.probefinder.com/roche3.html*); built on reference sequences available at NCBI (Table 1). *AGER* gene primer pairs were designed to detect one common amplicon of all mRNA splicing variants, which spans two exons, and then it was tested *in silico*. 

Two microliters of cDNA were amplified in 10 μl of a qPCR reaction mixture containing 1x FastStart Essential DNA Probes Master Mix (Cat. 06402682001), 400 nM of forward and reverse primers, and 200 nM of UPL probe. Reaction mixture was finally amplified in a LightCycler 96 (Roche Diagnostics, Germany) and the steps included the initial denaturation at 95 ^°^C for 10 min, followed by 45 cycles of denaturation at 95 ^°^C for 10 sec, annealing at 60 ^°^C for 30 sec, extension at 72 ^°^C for 1 sec, and an ending cooling step at 37 ^°^C for 30 sec.

Relative expression of the genes of interest was analyzed using the equation: Ratio=E_Target_^Δ^^Cp^_Target_^(control-sample)^/E_Ref_^Δ^^Cp^_Ref_^(control-sample) ^(11) and represented in arbitrary units. The *TBP* gene (Roche Diagnostics, Germany; Cat. 05 189 284 001), was used as a reference gene. *GAPDH* (Roche Diagnostics, Germany; Cat. 05 190 541 001) was used as an inducible gene expression variable control. 

*Protein extraction and Western blot analysis*

Total protein from PBMCs samples was prepared with T-Per (Pierce Chemical, USA; Cat. 78510) lysis solution with a protease inhibitor cocktail (Thermo Fisher Scientific, USA) and 1 mM of *phenylmethylsulfonyl* fluoride. Protein concentration was determined using the BCA protein assay kit (Thermo Fisher Scientific, USA; Cat. 23227). SDS-polyacrylamide gel (10%) electrophoresis was performed using 20 μg of protein per lane and transferred to an Immobilon-P transfer membrane (Millipore Co., USA, 0.45 μm). 

The membranes were blocked 1 hr at 4^ °^C with 5% non-fat dry milk in *Tris-buffered saline* (TBS) containing 0.1% Tween-20, before incubating overnight at 4 ^°^C with primary antibodies: polyclonal rabbit RAGE, GAPDH (ab34764 and ab2255 respectively, dilution 1:1000; Abcam Ltd, UK), and β-actin (sc-8432, 1:500; Santa Cruz Biotechnology, Inc, USA). Protein loading differences were normalized with β-actin followed by one additional hour with the appropriate horseradish peroxidase-linked secondary antibody.

Detection of peroxidase was performed with the Chemiluminescent HRP Substrate kit (EMD Millipore, USA; Cat. WBKLS0500). For imaging and digitalization, the MicroChemi imaging system (DNR Bio-Imaging Systems Ltd) was used. Quantitative results were obtained by densitometric analysis using Image J Software (National Institutes of Health, Bethesda, MD, USA).

*Immunocytochemistry*

The PBMC pellet resuspended in saline solution was fixed and permeabilized in a smear preparation. Slides were incubated with peroxidase block (Bio SB, USA) for 10 min. Non-specific protein binding was blocked with 1% bovine serum albumin for 1 hr.

Slides were incubated overnight in a humidified chamber with RAGE antibody (1:100) (see Western Blot assay) followed by incubation for 1 hr with an anti-rabbit horseradish peroxidase conjugated polymer (Bio SB, USA). 3,39-diaminobenzidine *versus* hematoxylin was used for immunostaining. Specific immuno-labeling was verified by negative control slides, which were omitting the primary antibody. A positive result was assigned when coffee staining was found in PBMC (either diffuse cytoplasmic stain or strong membranous strain) by two independent observers. Positive staining was documented on 200 cells of each sample and results were calculated in percentages.

***Statistical analysis***

The sample size obtained in this study was calculated based on the expression of RAGE in human PBMC estimated by Su *et al*. (12) to determine differences between the means and standard deviation of two independent samples, with a statistical power of 1−β= 0.8, a *P<*0.05 was considered as statistically significant. Quantitative data are expressed as the mean ± standard error of the media (SEM) and qualitative findings as percentages. Differences between groups were analyzed using the X^2^ test or one-way analysis of variance (ANOVA), as appropriate. After assuming variances as equal and not equal, the *post hoc* Bonferroni or T3 of Dunnett tests were applied respectively. Correlations between continuous variables were assessed using the Spearman Rank test. The statistical analysis was performed using SPSS 20.0 for Windows (SPSS Inc., Chicago, IL, USA).

## Results

***Clinical and biochemical parameters of the study groups***

Anthropometric and biochemical characteristics of the analyzed population are shown in Table 2. Mean age was 38.4±1.8 years, and 56% were females without statistically significant differences between groups. The anthropometric variables, such as fasting plasma glucose, insulin levels, and the HOMA-IR index of the OB-IR individuals were higher compared with HS and OB subjects. HbA1c levels were according to the IFCC criteria for IR (5.7 – 6.4%; NGSP-IFCC, 2010) even though they were not statistically significant. Moreover, serum levels of TG, VLDL, hs-CRP, and AST were also higher in subjects of the OB-IR group than in HS or OB groups (*P<*0.05). Hypertriglyceridemia and hypoalphalipoproteinemia were the most common dyslipidemias in the OB-IR group. Serum level of total sRAGE in subjects with OB-IR was lower than in the HS group (*P<*0.05), but MCP-1 levels were not different between groups.

**Figure 1:**
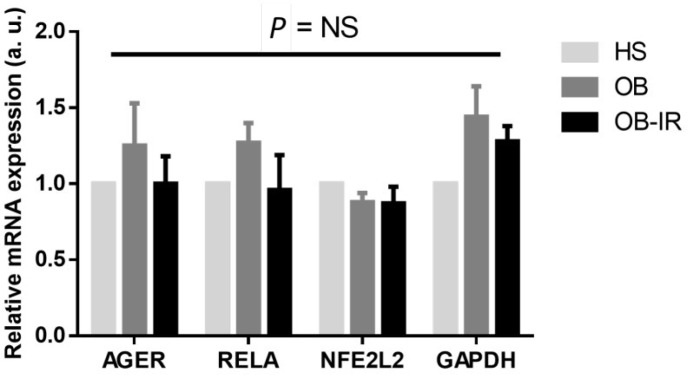
mRNA and protein expression in PBMC. The AGER, RELA, NFE2L2 and GAPDH gene expression of total sampling was evaluated by RT-qPCR of 1 µg of total RNA isolated from PBMC. Lean, healthy subjects (HS, n= 20), obese individuals (OB, n=20), and obese subjects with IR (OB-IR, n=17). Expression levels were normalized with the TBP gene. Mean±SEM corresponds to a.u., arbitrary units. NS= No Significance

**Figure 2:**
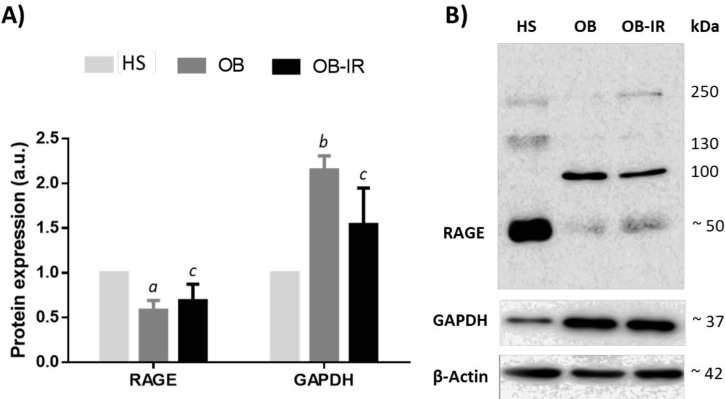
Comparisons of total protein lysates were analyzed in PBMC using Western blot. A) The bar graphs show decreased densitometric signal of 50 kDa RAGE protein and increased GAPDH protein expression in OB-IR and OB as compared to the HS group. B) Representative and complete blot of RAGE and GAPDH are shown. Samples (n=9 for each group) were normalized with respective densities of β-actin bands. Results are shown as fold change representing a relative expression according to the levels of expression in the HS group. Mean±SEM corresponds to a.u., relative arbitrary units

a Significance was from HS group (*P*<0.05)

b Significance was from HS group (*P*<0.001)

c No significance was from HS or OB group (*P*=NS)

**Figure 3:**
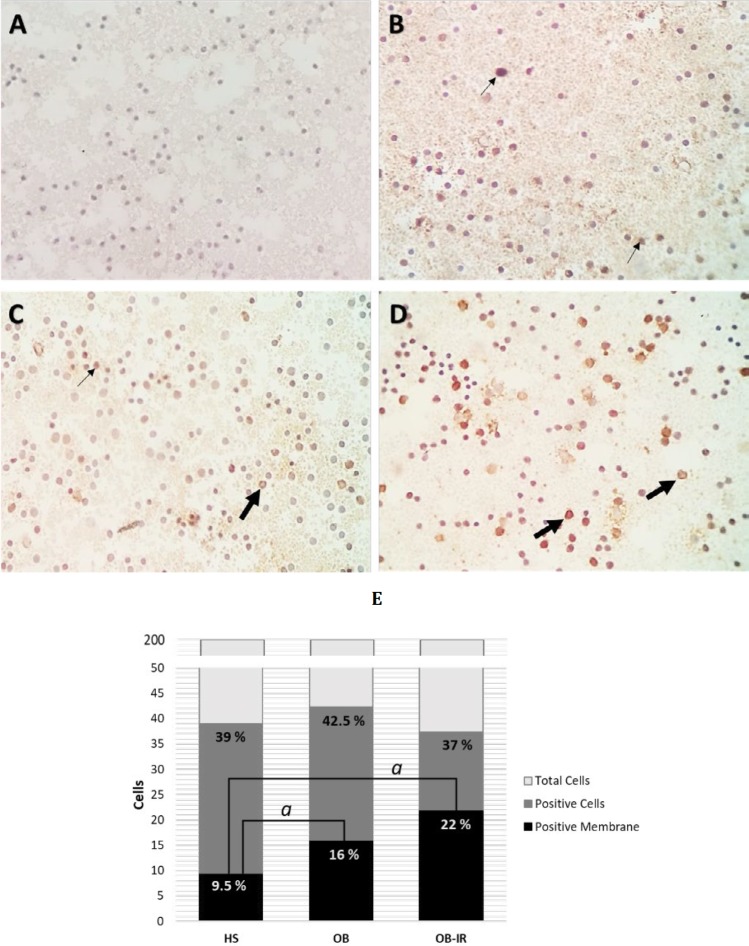
Distribution of RAGE protein expression in PBMC. PBMC slides were stained with anti-human RAGE. 40X Original magnification. Samples were: A) Negative control, B) Lean healthy subjects (HS), C) Obese subjects (OB) and D) obese and insulin resistant individuals (OB-IR). The distribution of RAGE was intracellular and on cell-surface, and these are marked with thin and thick arrows, respectively. E) Positive RAGE quantification of subcellular distribution. Data are expressed as percentage of 200 cells count by preparation (n=6 for each group). Data were analyzed using X2, *P*<0.001. The localization of RAGE is identified predominantly on the membrane in OB-IR group

**Table 1:** Primers of real time qPCR

Reference sequence	Gen	Protein	Primer	Product size (bp)	Probe
NM_001206966.1	*AGER*	RAGE	F: 5´-ggCAgACAgAgCCAggAC-3’	70	#38
			R: 5´-AgCACCCAggCTCCAACT-3’		
NM_001145413.1	*NFE2L2*	NRF2	F: 5’-TgTggCATCACCAgAACACT-3’	109	#52
			R: 5’-AggCCAAgTAgTgTgTCTCCA-3’		
NM_001243985.1	*RELA*	NF-kB (p65 subunit)	F: 5’-ACTgTgTgACAAggTgCAgAA-3’	103	#64
			R: 5’-CACTTgTCggTgCACATCA-3’		

Note: RAGE, receptor of advanced glycation end products; NRF2, nuclear factor erythroid 2-related factor 2; NF-kB, nuclear factor *kappa* B.

**Table 2:** Clinical and demographic characteristics of lean, healthy subjects (HS) with obesity (OB) and obesity plus insulin resistance (OB-IR). Data expressed as mean ± SEM.; except for gender that is expressed in absolute values and percentage. Comparison between groups is a= HS *vs* OB, b= HS *vs* OB-IR, c= OB *vs* OB-IR. Significant difference *P<*0.05. NS= Not significant

N	HS			OB			OB-IR			*P*-value
20			20			17		
Gender Male n (%) / Female n (%)	8 (40%) / 12 (60%)			9 (45%) / 11 (55%)			6 (35%) / 7 (65%)			NS
Age (years)	37.5	±	1.9	39.5	±	1.9	38.2	±	2.5	NS
Height (mt)	1.7	±	0.0	1.7	±	0.0	1.6	±	0.0	NS
Weight (kg)	64.8	±	1.9	93.4	±	2.5	93.5	±	3.6	a, b
BMI (kg/m^2^)	23.1	±	0.3	33.4	±	0.5	34.0	±	1.2	a, b
Hip (cm)	96.2	±	1.5	115.0	±	1.3	115.2	±	2.9	a, b
Waist (cm)	79.8	±	1.7	105.4	±	2.7	109.3	±	2.3	a, b
Waist – Hip index	0.8	±	0.0	0.9	±	0.0	1.0	±	0.0	a, b
Fat Free Mass (kg)	48.2	±	2.9	52.4	±	2.8	49.0	±	3.9	NS
Body Fat Mass (kg)	16.3	±	1.5	37.5	±	1.8	39.8	±	2.6	a, b
Body Fat Mass (%)	25.4	±	1.5	37.5	±	1.8	43.7	±	1.8	a, b
Fasting insulin (pmol)	2.9	±	0.3	4.5	±	0.3	16.6	±	1.7	a, b, c
Fasting glucose (mg/dL)	84.1	±	3.0	92.6	±	3.0	113.2	±	7.4	b, c
HOMA- IR	0.6	±	0.1	1.0	±	0.1	4.4	±	0.4	b, c
HbA1c (%)	5.1	±	0.2	5.6	±	0.4	5.7	±	0.6	NS
hs-CRP (mg/L)	2.9	±	2.1	5.9	±	0.9	7.3	±	1.2	a, b
sRAGE [pg/mL]	1185	±	93.8	921.6	±	68.1	913.4	±	53.8	a, b
MCP-1 [pg/mL]	311.0	±	35.0	290.5	±	15.4	346.9	±	28.6	NS
Cholesterol (mg/dL)	145.4	±	6.6	161.9	±	5.3	181.9	±	12.7	NS
Triglycerides (mg/dL)	96.5	±	7.9	124.2	±	15.1	191.6	±	27.8	b
HDL-c (mg/dL)	42.8	±	2.7	39.7	±	2.7	34.7	±	2.0	NS
LDL-c (mg/dL)	86.5	±	6.3	96.4	±	5.3	108.9	±	10.0	NS
VLDL-c (mg/dL)	19.3	±	1.6	25.2	±	3.1	38.4	±	5.5	b
AST (U/L)	25.9	±	3.2	22.6	±	1.8	36.7	±	4.7	c
ALT (U/L)	24.1	±	3.9	23.4	±	2.8	39.9	±	8.6	NS
ALPK (U/L)	94.4	±	8.4	111.9	±	10.3	95.5	±	8.1	NS
GGT (U/L)	25.3	±	4.6	31.3	±	4.6	54.9	±	22.1	NS
MetS n (%)	0 (0%)			4 (20%)			15 (88%)			b, c

***mRNA expression of AGER, RELA, NF2L2, and GAPDH genes on PBMC***

Figure 1 shows the mRNA expression of *AGER, RELA, NF2L2,* and *GAPDH* genes in PBMC. There is a higher average of the relative expression of mRNA of *AGER* and *RELA* in OB subjects regarding the control. On the other hand, *NFE2L2* tends to be lower in OB and OB-IR subjects. Higher *GAPDH* was also found in OB and OB-IR groups. These slight changes were not significant.

***RAGE and GAPDH protein***

To examine the impact of mRNA expression on protein levels normalized with respect to β-actin, we measured lysates in PMBC by Western Blot analysis. The antibody against RAGE displayed immunodetection at several molecular weights. RAGE-fl corresponds to a band of ~50 kDa. In the OB and OB-IR groups, RAGE expression was lower in comparison to the HS group, (0.58±0.11; *P*=0.034 and 0.68±0.18;* P=*NS, fold change, respectively), see Figure 2-A, B. PBMC protein lysates of the OB and OB-IR subjects had higher GAPDH (2.15±0.16 and 1.54±0.41-fold change, respectively), where a statistically significant difference was found on the OB versus the HS group (Figure 2). The expression profile of *GAPDH* mRNA was confirmed at protein levels in contrast to the lack of congruence between mRNA and protein expression of RAGE in PBMC.

***RAGE localization***

We also analyzed the presence and the subcellular distribution of RAGE in PBMC smears, using the same antibody as in the Western Blot assays (see the methods section). Positive immunoreaction was indicated by stained cells, and these counted cells were membrane-localized. A higher RAGE detection in the number of PBMC in OB as compared to the HS group was found; however, this difference was not significant (42.5% *vs* 39%, *P=*NS). Interestingly, the location was pronounced in the membrane in OB-IR subjects compared to the HS group (22% *vs* 9.5%, *P<*0.001) (Figure 3).

***Correlation analysis***

Direct correlations were found in the serum increase of glucose and insulin levels, HOMA-IR, as well as markers of lipid and hepatic profile with the anthropometric findings, data are not shown. The HOMA-IR correlated with hs-CRP (r=0.367, *P=*0.011) and ALT levels (r= 0.361, *P=*0.003). In our study, no signiﬁcant relationship between HbA1c, triglycerides levels, and other variables was found.

Regarding circulating sRAGE, negative correlations with HOMA-IR and ALT levels were found (r= -0.374, *P=*0.004; r= -0.429, *P=*0.001, respectively). In addition, sRAGE serum levels correlated negatively with mRNA relative expression of *AGER* (r= -0. 298, *P*=0.024) and positively with *NFE2L2* (r= 0.540, *P=*0.002). Finally, we found a weak correlation between quantified mRNA levels of *AGER* with *RELA* (r = -0.306, *P=*0.020) and *NFE2L2* (r= 0.359, *P=*0.006) on the same cells.

## Discussion

The majority of the subjects in this study are 40-year-old women. In addition to this, we detected that 82% of OB-IR group and the 24% of OB group had MetS determined by the presence of obesity, dyslipidemia**, **and hypertension. Diagnosis of IR was determined with fasting HOMA-IR that evaluates mainly the maintenance of glucose homeostasis due to inhibition of hepatic gluconeogenesis and glycogenolysis (10, 13). Presence of hyperinsulinemia was the main feature in this sample, discarding chronic hyperglycemia supported by the concentrations of HbA1c and blood glucose <125 mg/dl. Fasting hyperinsulinemia has been postulated as the earliest stage that drives IR in obesity (13). Despite the above**,** we found a difference of expression of GAPDH in PBMC between study groups, being part of the glycolytic pathway. Hence, the variations of GAPDH expression are sensitive to metabolic changes in these cells, in the same way, as in adipose tissue from OB-IR subjects (14). 

In this study, we found lower sRAGE serum levels in subjects with IR. This finding is consistent with previous reports of lowered sRAGE serum levels in obese subjects with several MetS components such as central obesity, hypertension, hypertriglyceridemia, and hypoalphaproteinemia (3, 15, 16) or have been related with an impaired glucose tolerant condition linked to the development of T2D (17); in addition, our observations are in accordance with those findings that suggest that sRAGE eliminates harmful ligands and acts as a competitive inhibitor of the ligands that bind to the cellular RAGE by attenuating the inflammatory cascade and thus, decreasing the sRAGE scavenger, increasing the risk of tissue damage (18). Furthermore, it was demonstrated that serum levels of sRAGE are down-regulated by hyperglycemia (19). All these data suggest that sRAGE response to early metabolic changes during the development of T2D in obese individuals.

To our knowledge, sRAGE levels reference values have not been established. Under normal conditions, sRAGE is produced mainly by proteolytic cleavage (65–70%) compared to esRAGE synthesis (30–35%) (20). Even though a study found that both esRAGE and sRAGE cleavage were lower in T2D and impaired-glucose-tolerance subjects compared to the HS group, they concluded that sRAGE cleavage is the principal isoform lost in the same groups (17). Protein ectodomain shedding made by metalloproteinases is part of a regulatory process and reflects an ongoing inflammation (21). Previous studies reported that proteolytic cleavage and expression might increase by insulin (22). 

The reduction of the protein ectodomain shedding of sRAGE may be a tissue-specific characteristic of the impairing signaling of IR. It is desirable that muscle, adipose, and liver could be main tissue sources driving regulation of sRAGE in this context; nevertheless, our work highlights some evidence of the process before the proteolytic cleavage of RAGE that involves membrane translocation in PBMC. Moreover, total sRAGE negatively correlates with liver biomarkers as ALT, and it is consistent with our findings; several studies have demonstrated that the presence of IR correlates positively with the increase in ALT (23). These findings suggest the increase of hepatic injury and low anti-inflammatory/oxidative defense mediated by sRAGE.

The evaluation of RAGE expression in PBMC has been focused on the presence of T2D (24), MetS (25), and in advanced aged individuals (26). In disagreement with our findings, these studies reported higher expression of *AGER*. 

Even though there were no significant changes in NF-kB and NRF2 mRNA expression in PBMC and serum MCP-1, patients showed a significant increase of hs-CRP levels as obesity and IR progress, manifesting the systemic inflammatory, and higher GGT levels were found indicating the pro-oxidative effect, being more sensitive than the expression of *NFE2L2*. Previous studies in PBMC indicate a lower expression of *NFE2L2* mRNA in subjects with T2D (27), and NRF2 protein in IR (28); researchers reported a negative correlation, where the increase of NRF2 activation attenuates RAGE protein in murine model with a high-fat diet supplemented with bioactive compounds: phloretin and/or gingerol (29). The strong positive correlation of sRAGE with mRNA expression of *NFE2L2* in PBMC, despite being slightly lower in individuals with obesity and IR, is consistent with the above. 

RAGE mRNA expression data manifested a different tendency at protein levels. The N-terminus domain is bound by the antibody used in this study, which resulted in a significant decrease of monomeric RAGE of 50 kDa (RAGE-fl isoform) in OB and OB-IR. In contrast to the levels of mRNA where RAGE relative expression showed a slightly higher in the OB group and primers were designed to include all isoforms. Although splicing variants and their products in specific tissues might also influence the pathogenesis mediated by RAGE, we suspect that another cause besides the differential expression of the isoforms could be the cause of the discrepancy of mRNA and protein levels, because RAGE-fl is recognized as the isoform that transduces the signal and is estimated to be equivalent to 80% of the total synthesis (30). A study similar to ours in design, also found a decrease in protein levels of RAGE in PBMC of OB-IR and HS compared to the OB group (31). Conversely, another study showed an increment in the RAGE protein levels in T cells from at-risk subjects who progressed to T1D (32), as well as an increase in the liver and subcutaneous and omental adipose tissues from obese non-diabetics and obese diabetics compared to non-obese subjects (33). It is important to mention that signals with higher molecular weight sizes were detected by the antibody used in this study, which could be a result of protein interaction and oligomerization (34). Further studies should include analysis on the regulation of isoforms of RAGE and its oligomerization to corroborate these hypotheses. Despite the limitations regarding the discrimination between the studied isoforms and protein interactions, suggests a dynamic regulation in RAGE expression.

We found a difference between ICC and Western Blot methods using the same antibody; while our data indicate that the number of cells stained is slightly higher in OB than in the OB-IR and HS groups, a significant RAGE protein decrease was found in OB and OB-IR versus the HS group. However, the main finding is the change of localization from intracellular to membranous RAGE protein. Hadding *et al.* were among the first to demonstrate different subcellular localizations of RAGE (35); these changes could be associated with membrane oligomerization (34) and protein interaction. A study showed that transmembrane localization of RAGE is related to the presence of circulating ligands, mainly T cells CD11c + in subjects with T1D and T2DM (36). Most of the studies above were determined by flow cytometric analysis (31, 32, 36), our results were made by ICC which allowed us to discern the location on the membrane and cytoplasmatic RAGE. Likewise, previous findings have shown that interactions between intracellular RAGE and its endogenous ligand HMGB1 in adipose tissue, are also crucial in activating chronic inflammation in subjects with obesity (37).

Our study shows that the main change in PBMC of RAGE expression is the subcellular localization between OB and OB-IR subjects, where its cellular trafficking appears very active, and the membranal translocation mechanisms may be the most important and dynamic part of the pathway activation in these cells. Previous studies have found a co-localization of RAGE in endosomes using RhoB, a protein located in the plasma membrane and endosomes (36). Rho GTPases are molecules that regulate membranous traffic (38). In this context, we hypothesize that among more ligands collide with the cell will produce a higher translocalization effect of RAGE cytosolic migration to transmembrane. Previous studies have described that some cytokines activated small GTPase in fibroblasts, which may be involved in this process; also, the binding of RAGE ligands to their cell surface receptor allows activation of molecules as Rac-1 and Cdc42 which are implicated in intracellular protein trafficking and cellular migration (39).

There were also study limitations: due to the cross-sectional nature of the study design, causal inferences cannot be made. Dynamic assays for the evaluation of insulin resistance were not performed. Hence data should be established for the fasting phenotype of the entire insulin resistance spectrum. Participants were recruited from the general population; most of them were women. Further analysis is necessary to understand how all these mechanisms related to the RAGE location (intracellular or membranous) are linked with inflammation and oxidative stress in the early stages of obesity and its complications. 

## Conclusion

Our results showed that correlations detected between sRAGE, biochemical parameters, and NRF2, besides the predominant RAGE distribution on the cell membrane in PBMC could be evidence of the early phase of the inflammatory cascade and the subsequent damage in specific tissues in subjects with OB-IR.
